# Exploring the pathogenesis of colorectal carcinoma complicated with hepatocellular carcinoma via microarray data analysis

**DOI:** 10.3389/fphar.2023.1201401

**Published:** 2023-06-13

**Authors:** Tianqi Gao, Mengping Li, Dailin Wu, Ni Xiao, Dan Huang, Li Deng, Lunwei Yang, Chunhong Tian, Yang Cao, Jun Zhang, Jihong Gu, Yang Yu

**Affiliations:** ^1^ The First Clinical College of Guangzhou University of Chinese Medicine, Guangzhou, China; ^2^ Department of Oncology, The Affiliated TCM Hospital of Guangzhou Medical University, Guangzhou, China; ^3^ Department of Oncology, The First Affiliated Hospital of Guangzhou University of Chinese Medicine, Guangzhou, China; ^4^ Science and Technology Innovation Center, Guangzhou University of Chinese Medicine, Guangzhou, Guangdong, China; ^5^ Department of Gastrointestinal and Thyroid Surgery, The First Affiliated Hospital of Guangzhou University of Chinese Medicine, Guangzhou, China

**Keywords:** colorectal carcinoma, hepatocellular carcinoma, differentially expressed genes, hub genes, survival analysis

## Abstract

**Background:** Despite the increasing number of research endeavors dedicated to investigating the relationship between colorectal carcinoma (CRC) and hepatocellular carcinoma (HCC), the underlying pathogenic mechanism remains largely elusive. The aim of this study is to shed light on the molecular mechanism involved in the development of this comorbidity.

**Methods:** The gene expression profiles of CRC (GSE90627) and HCC (GSE45267) were downloaded from the Gene Expression Omnibus (GEO) database. After identifying the common differentially expressed genes (DEGs) of psoriasis and atherosclerosis, three kinds of analyses were performed, namely, functional annotation, protein‐protein interaction (PPI) network and module construction, and hub gene identification, survival analysis and co-expression analysis.

**Results:** A total of 150 common downregulated differentially expressed genes and 148 upregulated differentially expressed genes were selected for subsequent analyses. The significance of chemokines and cytokines in the pathogenesis of these two ailments is underscored by functional analysis. Seven gene modules that were closely connected were identified. Moreover, the lipopolysaccharide-mediated signaling pathway is intricately linked to the development of both diseases. Finally, 10 important hub genes were identified using cytoHubba, including CDK1, KIF11, CDC20, CCNA2, TOP2A, CCNB1, NUSAP1, BUB1B, ASPM, and MAD2L1.

**Conclusion:** Our study reveals the common pathogenesis of colorectal carcinoma and hepatocellular carcinoma. These common pathways and hub genes may provide new ideas for further mechanism research.

## Introduction

Globally, the incidence and mortality rates of colorectal cancer (CRC) continue to remain high. In 2020, worldwide statistics reported almost two million newly diagnosed cases and over 900,000 deaths, representing 10.0% and 9.4% of cancer incidence and mortality, respectively. Colorectal cancer ranks as the third and second most common malignant tumor in terms of incidence and mortality, respectively. Furthermore, there appears to be a gradual decrease in the age of onset (Sung et al., 2021). Accounting for approximately 80%–90% of all primary liver malignancies, primary hepatocellular carcinoma (HCC) ranks as the third most common cause of cancer-related deaths globally (Peeters and Dekervel, 2023). These two diseases seriously threaten the health of mankind.

Dual primary carcinoma is a type of multiple malignant tumors. It can be classified into synchronous carcinoma (SC) and metachronous carcinoma (MC), based on the time interval between the onset of the diseases, typically divided by using 6 months as the cutoff point (Park et al., 2005). In a study for 40 cases who confirmed by pathological examination as CRC combined with PHC double primary carcinoma suggested that both CRC and HCC belong to abdominal malignancies. However, since the liver is the main target organ for CRC hematogenous metastasis, imaging physicians tend to confuse the characteristics of CRC and HCC metastatic liver cancer in the identification of the characteristics of intrahepatic occupying lesions, as well as the coexistence of CRC and HCC liver metastases, it is difficult to make accurate diagnosis based on imaging data before surgery (Zhiping et al., 2023). Therefore, it is necessary to identify potential biomarkers and therapeutic targets for these coexisting metastases by other diagnostic tools.

Currently, there have been numerous reports of double primary cancer, where CRC is combined with HCC, both domestically and internationally. Approximately 15%–25% of patients with colorectal cancer present with synchronous liver metastases at the time of initial presentation (Kianmanesh et al., 2007; Engstrand et al., 2018). A retrospective cohort study demonstrated the risk for HCC with CRC, especially in patients with rectal cancer (Lee et al., 2015). A recent study showed that deoxycholic acid (DCA), a secondary bile acid known to cause DNA damage, may return to the liver through enterohepatic circulation and provoke the senescence-associated secretory phenotype in hepatic stellate cells, which in turn secretes pro-inflammatory and tumor-promoting factors in the liver and facilitates dual primary tumor development (Yoshimoto et al., 2013). Further studies are needed to explore this interesting phenomenon. However, few studies have been conducted to investigate the common pathway underlying these two diseases. The development of CRC and HCC has been linked to multiple altered signaling pathways. In CRC, about ninety percent of all tumors have a mutation in a key regulatory factor of the Wnt/β-catenin pathway, with APC or CTNNB1 being the most common affected genes. This mutation leads to the activation of the Wnt/β-catenin pathway (Wanitsuwan et al., 2008). HCC is also characterized by early genetic alterations in telomerase reverse transcriptase promoter (TERTp) and CTNNB1 genes and immune cell activation in the tumor microenvironment (Ambrozkiewicz et al., 2022).

The aim of this research is to discover hub genes related to the pathogenesis of colorectal carcinoma and hepatocellular carcinoma, and explore the potential common pathogenesis between the two diseases. To achieve this goal, two gene expression data sets (GSE90627 and GSE45267) were analyzed using comprehensive bioinformatics and enrichment analysis. The common differentially expressed genes (DEGs) and their functions in CRC and HCC were identified. Moreover, a protein-protein interaction (PPI) network was constructed to analyze gene modules and identify hub genes using the STRING database and Cytoscape software. The transcription factors of the hub genes were also analyzed and their expression was verified. Through this process, important hub genes and their prognostic value were analyzed. The discovery of these hub genes is expected to provide new insights into the biological mechanisms of CRC and HCC, particularly their shared transcription features.

## Materials and methods

### Data source

The GEO database (http://www.ncbi.nlm.nih.gov/geo) is a publicly accessible resource that contains numerous data sets obtained through high-throughput sequencing and microarray analyses. These data sets have been submitted by research institutions located all around the world (Edgar et al., 2002). To identify relevant gene expression datasets, we utilized the keywords CRC and HCC in our search. Our inclusion criteria specified that two distinct expression profiles originating from the same sequencing platform and featuring the largest sample size should be selected. Moreover, we only considered datasets that utilized human test specimens. Finally, two microarray datasets [GSE90627 (Guo et al., 2017) and GSE45267 (Chen et al., 2018)] were downloaded from it (Affymetrix GPL570 platform, Affymetrix Human Genome U133 Plus 2.0 Array). The GSE90627 dataset contains 32 paired patients with CRC and adjacent normal tissues. The GSE45267 dataset contains 74 paired patients with HCC and adjacent normal tissues. As all data were downloaded from public databases, ethics approval was not required due to no foreseeable impact on the rights and/or welfare of the subjects involved.

### Identification of DEGs

GEO2R (www.ncbi.nlm.nih.gov/geo/ge2r) is an online analysis tool developed based on 2 R packages (GEOquery and Limma) (Barrett et al., 2013). We employed the GEOquery package to access the data and performed differential expression analysis using the Limma package. To identify the DEGs between the control and diseased groups, we utilized GEO2R to compare gene expression profiles. Any probe sets lacking corresponding gene symbols or genes represented by more than one probe set were either excluded or averaged, respectively. The DEGs were limited to genes with adjusted *p*-value of <0.05 and |logFC (fold change) | values of ≥1. We used an online Venn diagram tool to identify common DEGs among the samples.

### Enrichment analyses of DEGs

The Gene Ontology (GO) database, established by the Gene Ontology Federation, provides functional annotations of gene products, identifies biological pathways, and specifies cell locations. Kyoto Encyclopedia of Genes and Genomes (KEGG) Pathway is a database that stores information on gene pathways in various species. KEGG Orthology Based Annotation System (KOBAS) is a web server for functional annotation and enrichment of genes and proteins that includes functional annotation data for 4325 species. We obtained the enrichment analysis results of GO and KEGG Pathway from the KOBAS 3.0 database (Wu et al., 2006). Adjusted *p*-value <0.05 was considered significant.

### PPI network construction and module analysis

Search Tool for the Retrieval of Interacting Genes (STRING; http://string-db.org) (version 11.0) can search for the relationship between proteins of interest, such as direct binding relationships, or coexisting upstream and downstream regulatory pathways, to construct a PPI network with complex regulatory relationships (Franceschini et al., 2013). Interactions with a combined score over 0.4 were considered statistically significant. Cytoscape (http://www.cytoscape.org) (version 3.7.2) was used to visualize this PPI network (Smoot et al., 2011). Cytoscape’s plug-in molecular complex detection technology (MCODE) was used to analyze key functional modules. Set the selection criteria as: K-core = 2, degree cutoff = 2, max depth = 100, and node score cutoff = 0.2. Then the KEGG and GO analysis of the involved modular genes were performed with KOBAS 3.0.

### Selection and survival analysis of hub genes

To identify the hub genes, we utilized the cytoHubba plug-in of Cytoscape and evaluated them using seven common algorithms, including MCC, MNC, Degree, Closeness, Radiality, Stress, and EPC. We selected the hub genes based on these evaluations. Next, we employed GeneMANIA (http://www.genemania.org/), a reliable tool for identifying internal associations in gene sets, to construct a co-expression network of these hub genes (Warde-Farley et al., 2010). The database also provides a large number of samples with a variety of clinical characteristics. The mRNA Seq data and clinical features of CRC and HCC were extracted from The Cancer Genome Atlas (TCGA) (https://www.cancer.gov/tcga) in August 2022 to perform a survival analysis, 698 cases of CRC and 424 cases of HCC were finally included. OS differences for two diseases were presented by K-M plots with log-rank *p*-values via TCGA by querying hub genes (Gao et al., 2013).

### Validation of hub genes expression in other data sets

The mRNA expression of identified hub genes was verified in GSE89076 (Satoh et al., 2017) and GSE50579 (Neumann et al., 2012). We conducted a comparison between the GSE89076 dataset, which comprises 275 paired patients with CRC and adjacent normal tissues, and the GSE50579 dataset, which consists of 73 paired patients with HCC and adjacent normal tissues. To determine the significance of the comparison, we employed the *t*-test, with *p*-value <0.05 considered statistically significant.

### Prediction and verification of transcription factors (TFs)

We utilized the Transcriptional Regulatory Relationships Unraveled by Sentence-based Text mining (TRRUST) database to predict transcriptional regulatory networks, which contains regulatory relationships between transcription factors (TFs) and target genes (Han et al., 2018). The database includes 8,444 and 6,552 TF-target regulatory relationships for 800 human and 828 mouse TFs, respectively. TFs regulating hub genes were identified using the TRRUST database and considered significant if adjusted *p*-value <0.05. We further validated the expression levels of these TFs using the *t*-test in GSE89076 and GSE50579 datasets.

## Results

### Identification of DEGs

**FIGURE 1:**
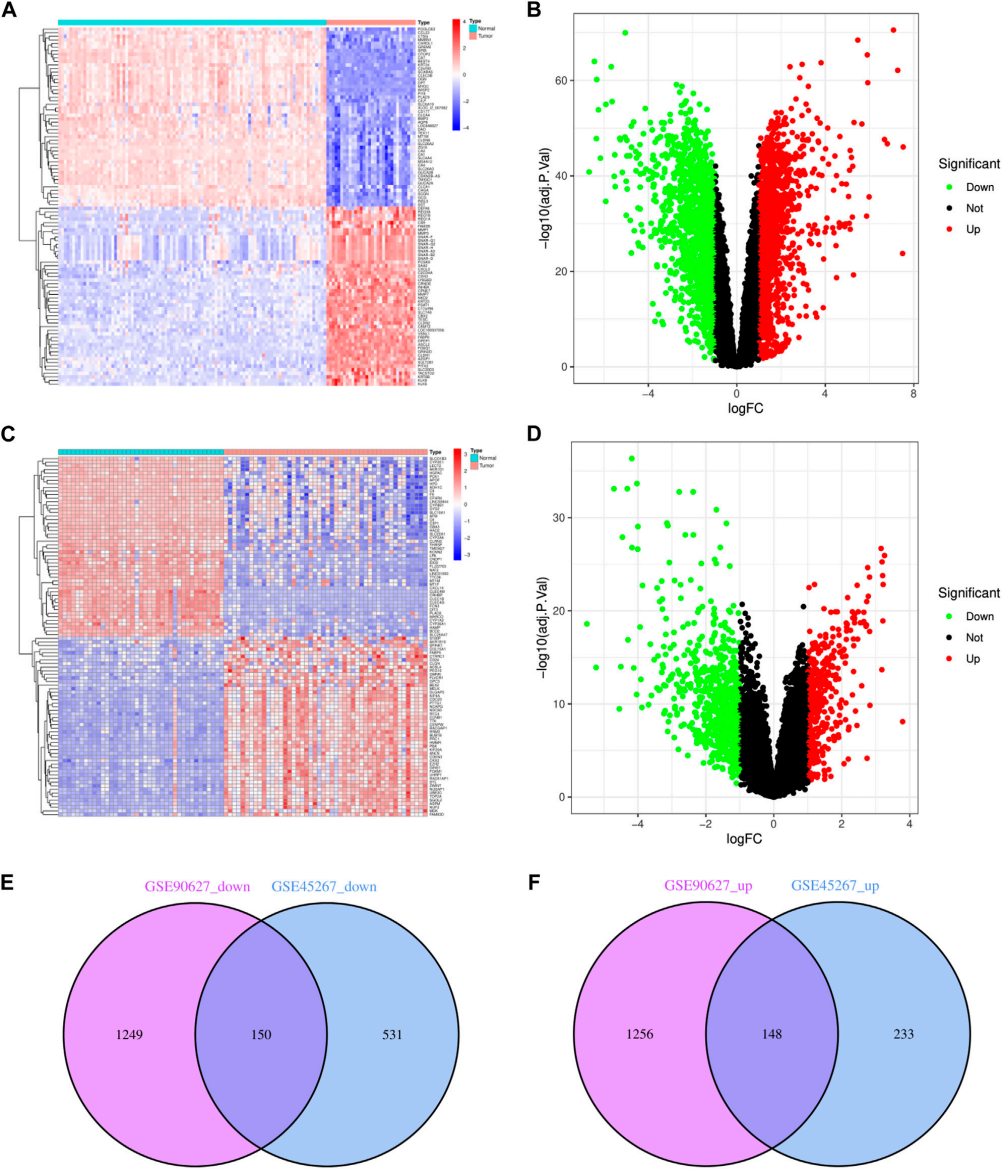
Identification of DEGs in CRC and HCC. **(A)** The heatmap of DEGs in GSE90627. **(B)** The volcano map of DEGs in GSE90627. **(C)** The heatmap of DEGs in GSE45267. **(D)** The volcano map of DEGs in GSE45267. **(E)** Overlap of 150 downregulated DEGs. **(F)** Overlap of 148 upregulated DEGs. In heatmap, upregulated genes are marked in light red; downregulated genes are marked in light blue; in volcano map, upregulated genes are marked in light red; downregulated genes are marked in light green.

Upon normalization of the microarray data, we were able to identify DEGs from GSE90627 (1399 DEGs) and GSE45267 (681 DEGs) (Figures 1A–D). By analyzing the intersection of the Venn diagram, we identified 150 commonly downregulated DEGs and 148 upregulated DEGs (Supplementary Table S1; Figures 1E, F).

### Analysis of the functional characteristics of common DEGs

**FIGURE 2:**
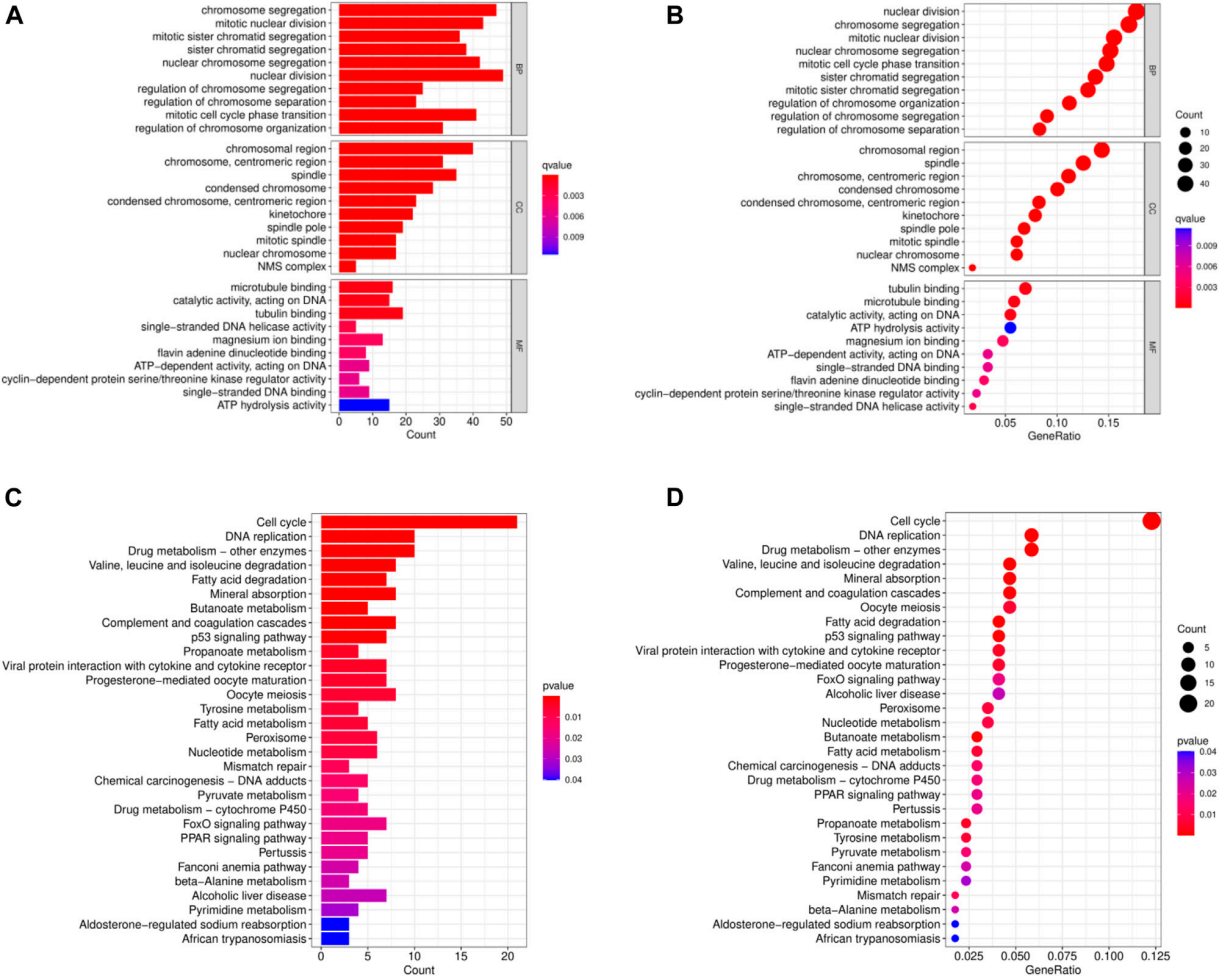
Common DEGs functional enrichment analysis results. **(A,B)** The enrichment analysis results of GO Pathway. **(C,D)** The enrichment analysis results of KEGG Pathway.

In order to analyze the biological functions and pathways involved in the 94 common DEGs, GO and KEGG Pathway enrichment analysis were performed. As showed in Figures 2A, B, GO analysis results show that the top 3 BP terms were “nuclear division”, “sister chromatid segregation” and “nuclear chromosome segregation”. The top 3 CC terms were “spindle”, “spindle pole” and “mitotic spindle”. The top 3 MF terms were “protein C-terminus binding”, “cyclin-dependent protein serine/threonine kinase regulator activity” and “histone deacetylase binding”. (All adjusted *p*-value <0.05). The KEGG Pathway analysis revealed that the enriched pathways for these two cancers were cell cycle, progesterone-mediated oocyte maturation, and oocyte meiosis, with all having an adjusted *p*-value <0.05 (Figures 2C, D). These findings suggest that cell cycle regulation and cytokines have a significant role in the development and progression of these two cancers, which was consistent with previous report.

### PPI network construction and module analysis

**FIGURE 3:**
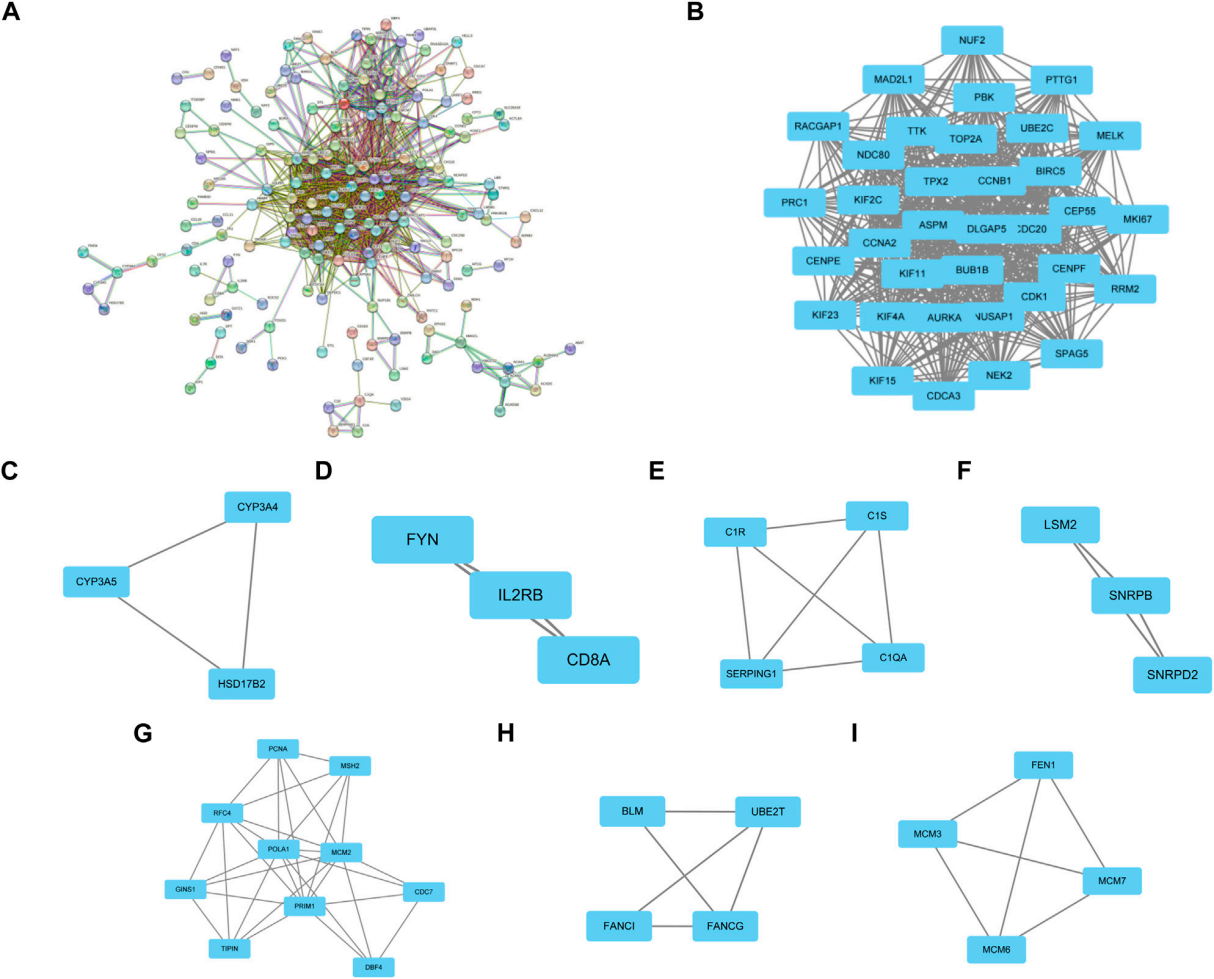
PPI network construction and significant gene module of the modular genes. **(A)** PPI network of co-expressed genes. **(B–I)** Eight significant gene clustering modules of 1089 pairs of interactions.

PPI networks of the common DEGs with combined scores were generated using the Gene Ontology string database and the Kyoto Encyclopedia of Genes and Genomes. The network in Figure 3A shows genes that are co-expressed. By using the MCODE plug-in of Cytoscape, we were able to identify seven gene modules that were closely connected. These modules included 66 common DEGs and 1089 pairs of interactions, as shown in Figures 3B–I.

### Selection and analysis of hub genes

**FIGURE 4:**
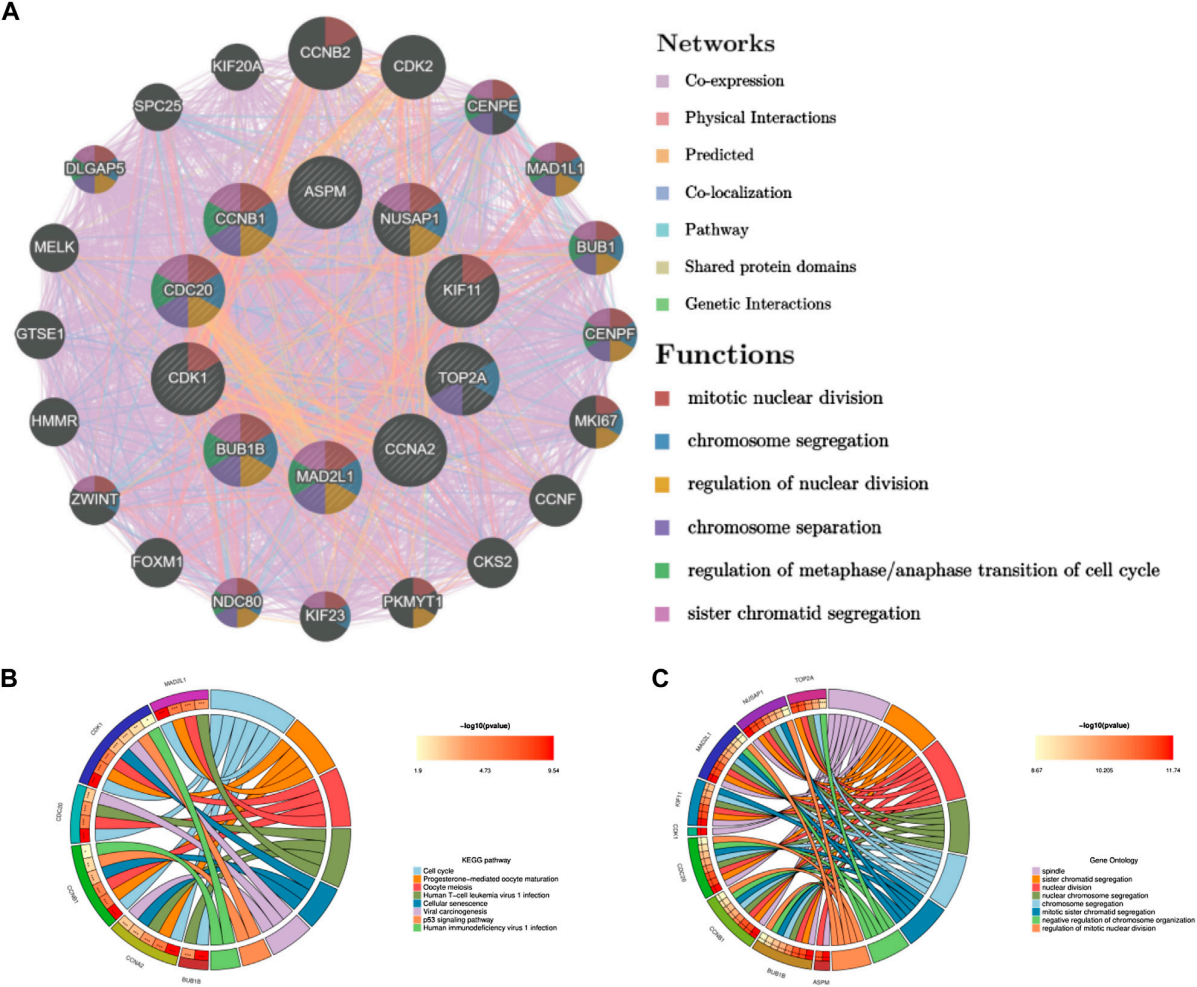
Co-expression network and functional analysis of hub genes. **(A)** Hub genes and their co-expression genes were analyzed via GeneMANIA. **(B)** GO enrichment analysis of the hub genes. **(C)** KEGG enrichment analysis of the hub genes.

Through the seven algorithms of plug-in cytoHubba, we have calculated the top 30 hub genes (Table 1), After taking the intersection of the Venn diagrams, we found 10 common important hub genes, including CDK1, KIF11, CDC20, CCNA2, TOP2A, CCNB1, NUSAP1, BUB1B, ASPM and MAD2L1 (Figure 4A). We utilized the GeneMANIA database to investigate the co-expression network and associated functions of the genes. These genes showed the complex PPI network with the co-expression of 56.49%, physical interactions of 17.39%, colocalization of 7.75%, predicted of 14.91% and pathway of 3.44%. The GO analysis of these genes (Figure 4B) indicated their predominant involvement in spindle, sister chromatid segregation, and nuclear division. Moreover, the KEGG Pathway analysis (Figure 4C) demonstrated their significant involvement in cell cycle, progesterone-mediated oocyte maturation, and oocyte meiosis. These findings underscore the crucial role of cytokines and cell cycle regulation in the pathogenesis of the two diseases.

**TABLE 1:** The details of the top 30 hub genes.

No.	Gene symbol	Full name	Function
1	ASPM	assembly factor for spindle microtubules	This gene is essential for normal mitotic spindle function in embryonic neuroblasts
2	NUSAP1	nucleolar and spindle associated protein	NUSAP1 is a nucleolar-spindle-associated protein that plays a role in spindle microtubule organization
3	KIF11	kinesin family member 11	This gene encodes a motor protein that belongs to the kinesin-like protein family which are known to be involved in various kinds of spindle dynamics
4	TOP2A	DNA topoisomerase II alpha	This gene encodes a DNA topoisomerase, an enzyme that controls and alters the topologic states of DNA during transcription
5	CCNA2	cyclin A2	The protein encoded by this gene belongs to the highly conserved cyclin family, whose members function as regulators of the cell cycle
6	MAD2L1	mitotic arrest deficient 2 like 1	MAD2L1 is a component of the mitotic spindle assembly checkpoint that prevents the onset of anaphase
7	BUB1B	BUB1 mitotic checkpoint serine/threonine kinase B	This gene encodes a kinase involved in spindle checkpoint function
8	CDK1	cyclin dependent kinase 1	The protein encoded by this gene is a member of the Ser/Thr protein kinase family which is essential for G1/S and G2/M phase transitions of eukaryotic cell cycle
9	CDC20	cell division cycle 20	CDC20 appears to act as a regulatory protein interacting with several other proteins at multiple points in the cell cycle
10	CCNB1	cyclin B1	The protein encoded by this gene is a regulatory protein involved in mitosis
11	CCNB2	cyclin B2	Cyclin B2 is a member of the cyclin family which are essential components of the cell cycle regulatory machinery
12	CDK2	cyclin dependent kinase 2	This gene encodes a member of a family of serine/threonine protein kinases that participate in cell cycle regulation
13	CENPE	centromere protein E	Centrosome-associated protein E (CENPE) is a kinesin-like motor protein that accumulates in the G2 phase of the cell cycle
14	MAD1L1	mitotic arrest deficient 1 like 1	MAD1L1 is a component of the mitotic spindle-assembly checkpoint and may play a role in cell cycle control and tumor suppression
15	BUB1	BUB1 mitotic checkpoint serine/threonine kinase	This gene encodes a serine/threonine-protein kinase that play a central role in mitosis and also plays a role in inhibiting the activation of the anaphase promoting complex/cyclosome
16	CENPF	centromere protein F	This gene encodes a protein that associates with the centromere-kinetochore complex.It play a role in chromosome segregation during mitotis
17	MKI67	marker of proliferation Ki-67	Enables protein C-terminus binding activity. Involved in regulation of chromosome segregation and regulation of mitotic nuclear division
18	CCNF	cyclin F	This gene encodes a member of the cyclin family. Cyclins are important regulators of cell cycle transitions
19	CKS2	CDC28 protein kinase regulatory subunit 2	CKS2 protein binds to the catalytic subunit of the cyclin dependent kinases and is essential for their biological function
20	PKMYT1	protein kinase, membrane associated tyrosine/threonine 1	This gene encodes a member of the serine/threonine protein kinase family. The encoded protein is a membrane-associated kinase
21	KIF23	kinesin family member 23	The protein encoded by this gene is a member of kinesin-like protein family
22	NDC80	NDC80 kinetochore complex component	This gene encodes a component of the NDC80 kinetochore complex. This protein functions to organize and stabilize microtubule-kinetochore interactions
23	FOXM1	forkhead box M1	The protein encoded by this gene is a transcriptional activator involved in cell proliferation
24	ZWINT	ZW10 interacting kinetochore protein	This gene encodes a protein that is clearly involved in kinetochore function
25	HMMR	hyaluronan mediated motility receptor	The protein encoded by this gene is involved in cell motility. It is expressed in breast tissue
26	GTSE1	G2 and S-phase expressed 1	The protein encoded by this gene is only expressed in the S and G2 phases of the cell cycle.The encoded protein binds the tumor suppressor protein p53, repressing its ability to induce apoptosis
27	MELK	maternal embryonic leucine zipper kinase	Enables calcium ion binding activity.Involved in apoptotic process; cell population proliferation; and protein autophosphorylation
28	DLGAP5	DLG associated protein 5	Enable microtubule binding activity. Involved in several processes, including centrosome localization; kinetochore assembly; and mitotic spindle organization
29	SPC25	SPC25 component of NDC80 kinetochore complex	This gene encodes a protein that involved in kinetochore-microtubule interaction and spindle checkpoint activity
30	KIF20A	kinesin family member 20A	Enables protein kinase binding activity. Involved in microtubule bundle formation; midbody abscission; and regulation of cytokinesis

### Validation and survival analysis of hub genes expression in two diseases

**FIGURE 5:**
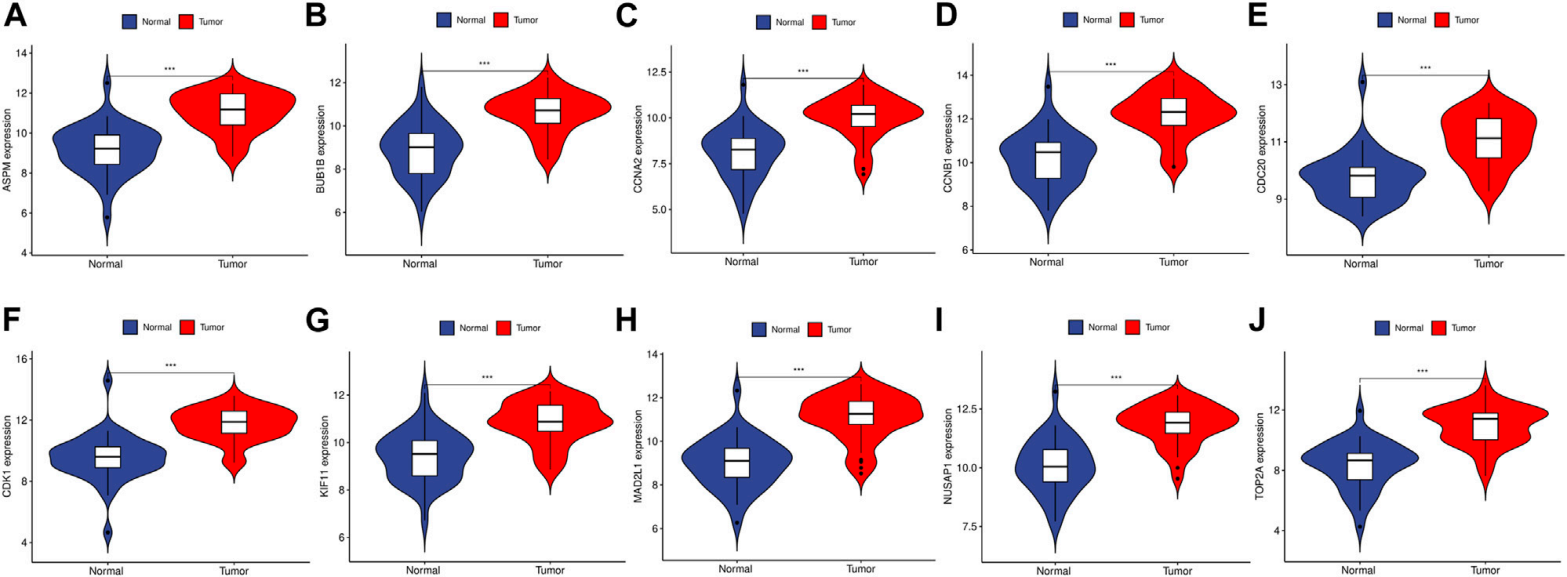
The relative expression level of hub genes in GSE89076. **(A–J)** Expression level of ASPM, BUB1B, CCNA2, CCNB1, CDC20, CDK1, KIF11, MAD2L1, NUSAP1 and TOP2A, respectively. The upregulated genes are marked in light red; downregulated genes are marked in light blue. The comparison between the two sets of data uses the mean *t*-test. *p*-value <0.05 was considered statistically significant. ***p < 0.001.

**FIGURE 6:**
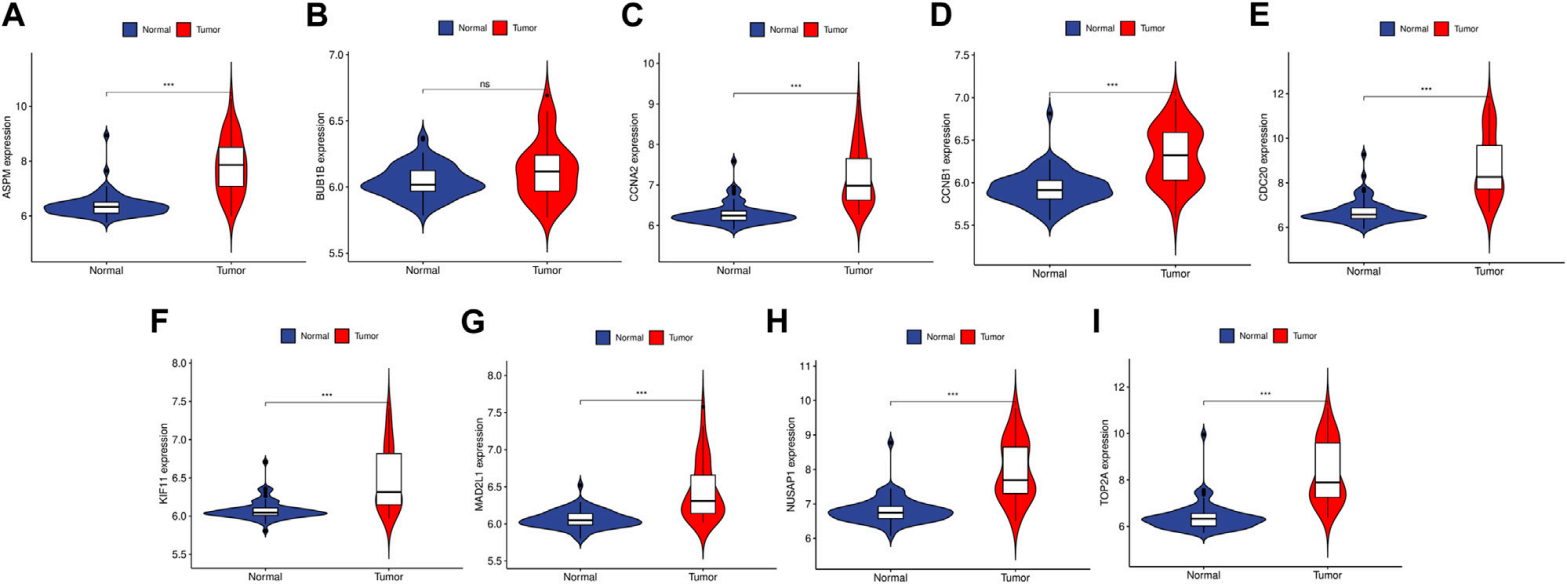
The relative expression level of hub genes in GSE50579. **(A–I)** Expression level of ASPM, BUB1B, CCNA2, CCNB1, CDC20, KIF11, MAD2L1, NUSAP1 and TOP2A, respectively. The upregulated genes are marked in light red; downregulated genes are marked in light blue. The comparison between the two sets of data uses the mean *t*-test. *p*-value <0.05 was considered statistically significant. ***p < 0.001.

To validate the reliability of the expression levels of these hub genes, we analyzed their expression levels in two additional datasets of HCC and CRC. The results indicated that in comparison to the normal tissue adjacent to the tumor tissue, all hub genes were significantly upregulated in CRC (Figure 5) and eight genes were upregulated in HCC (Figure 6).

**FIGURE 7:**
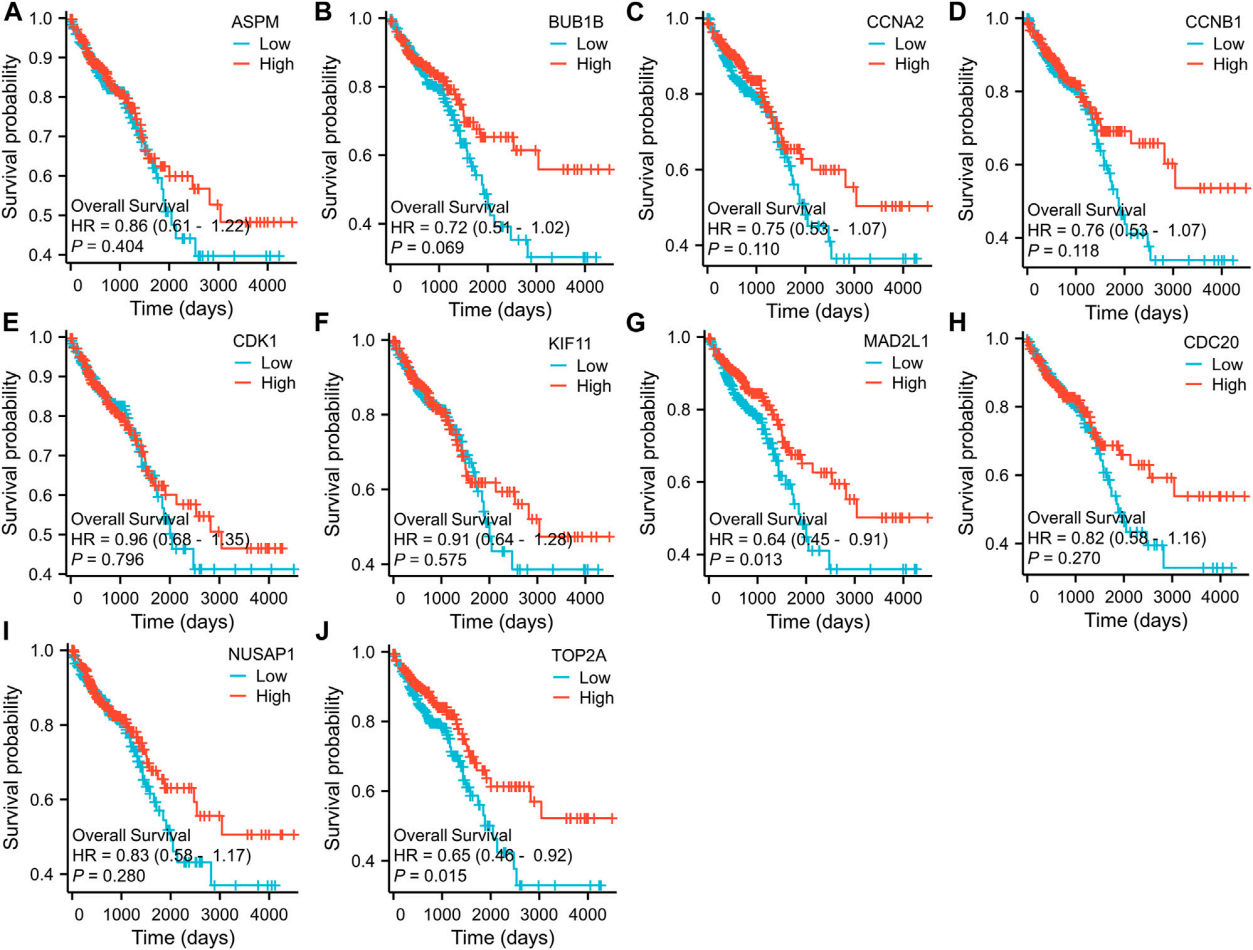
Prognostic significance of hub genes expression in CRC patients. **(A–J)** Kaplan−Meier curves of survival analysis for ASPM, BUB1B, CCNA2, CCNB1, CDC20, CDK1, KIF11, MAD2L1, NUSAP1 and TOP2A, respectively.

**FIGURE 8:**
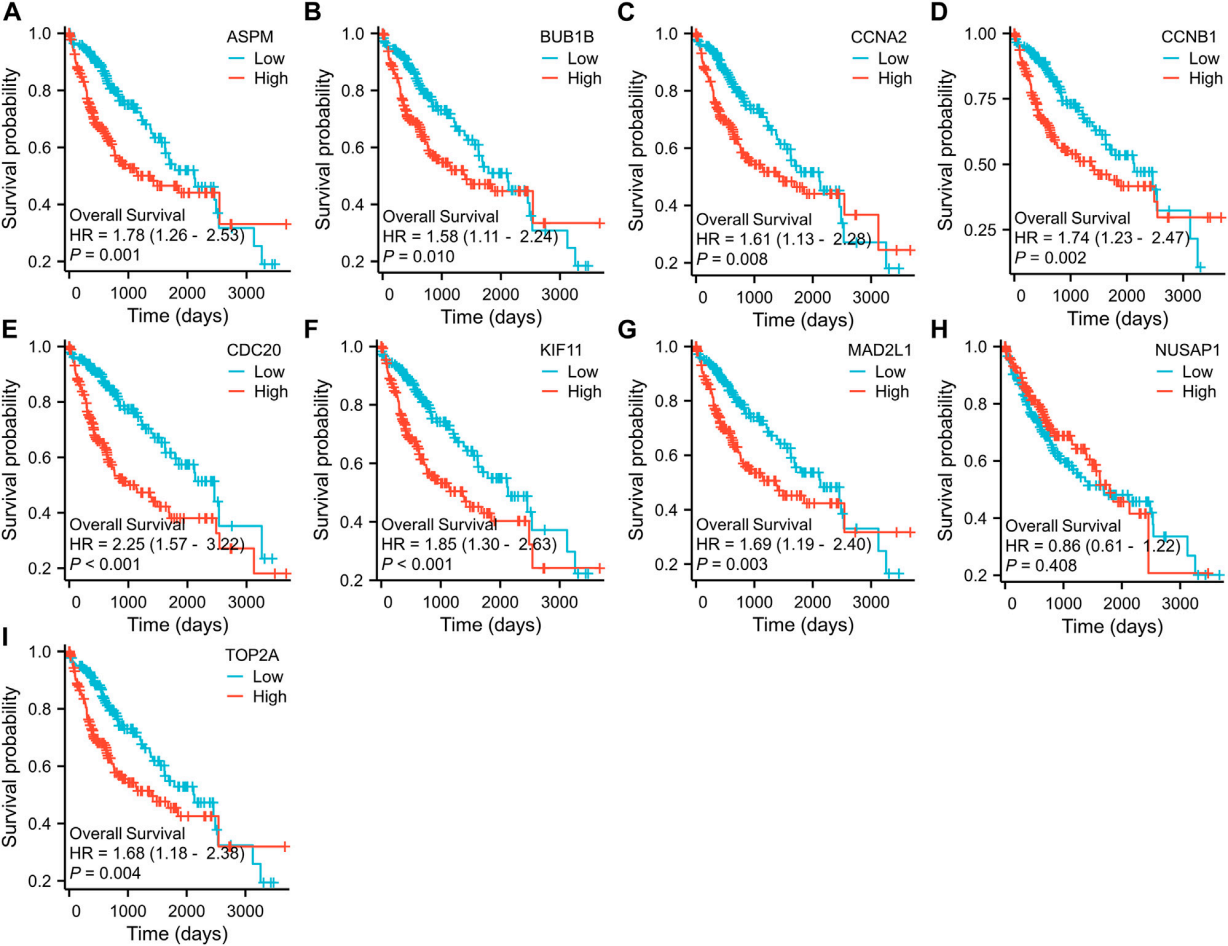
Prognostic significance of hub genes expression in HCC patients. **(A–I)** Kaplan−Meier curves of survival analysis for ASPM, BUB1B, CCNA2, CCNB1, CDC20, KIF11, MAD2L1, NUSAP1 and TOP2A, respectively.

To further examine the correlation between the expression of these hub genes and the clinical outcomes in these two diseases, we performed Cox regression survival analyses using the Kaplan-Meier method based on the TCGA database. The results revealed that patients with CRC in the low expression groups of MAD2L1 and TOP2A exhibited significantly decreased overall survival (OS) compared to those in the high expression groups (*p* = 0.013 and 0.015, respectively) (Figure 7). Conversely, patients with HCC in the low expression groups of ASPM, BUB1B, CCNA2, CCNB1, CDC20, KIF11, MAD2L1, and TOP2A demonstrated significantly better OS than those in the high expression groups (all *p* < 0.05) (Figure 8). Remarkably, high expression of these hub genes appears to have opposite prognostic implications in CRC and HCC.

### Prediction and verification of TFs

**FIGURE 9:**
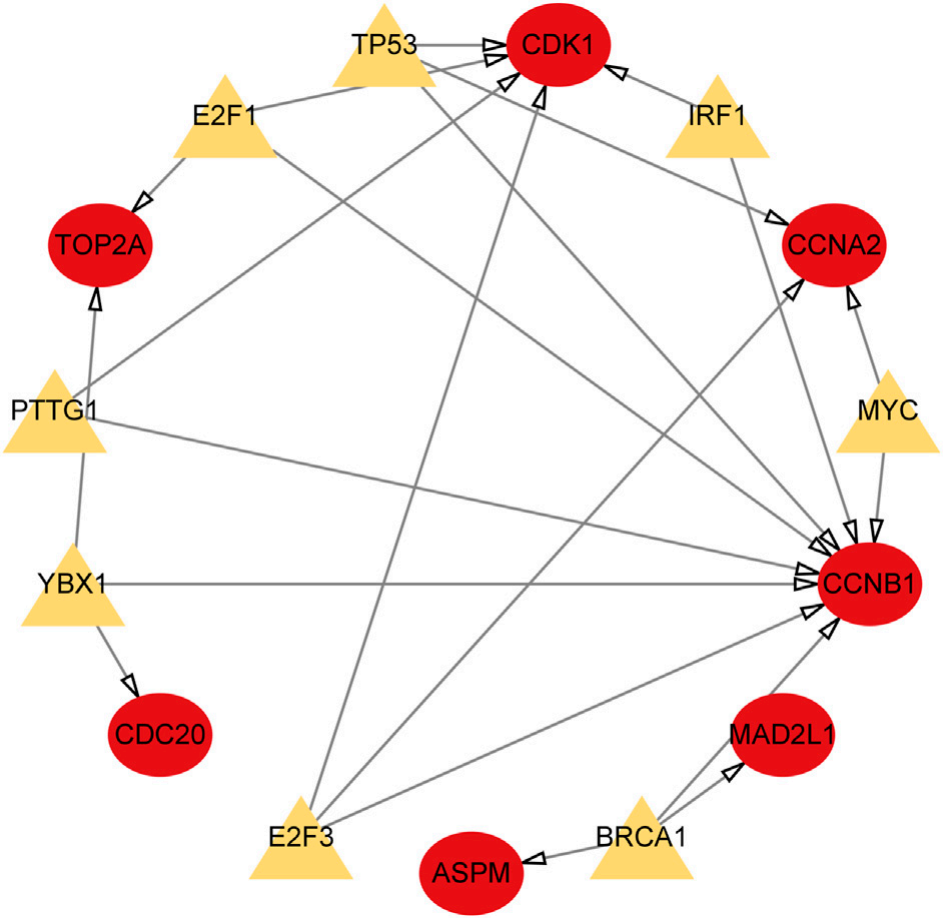
TFs regulatory network. TFs were marked in yellow, and the hub genes were marked in red.

**FIGURE 10:**
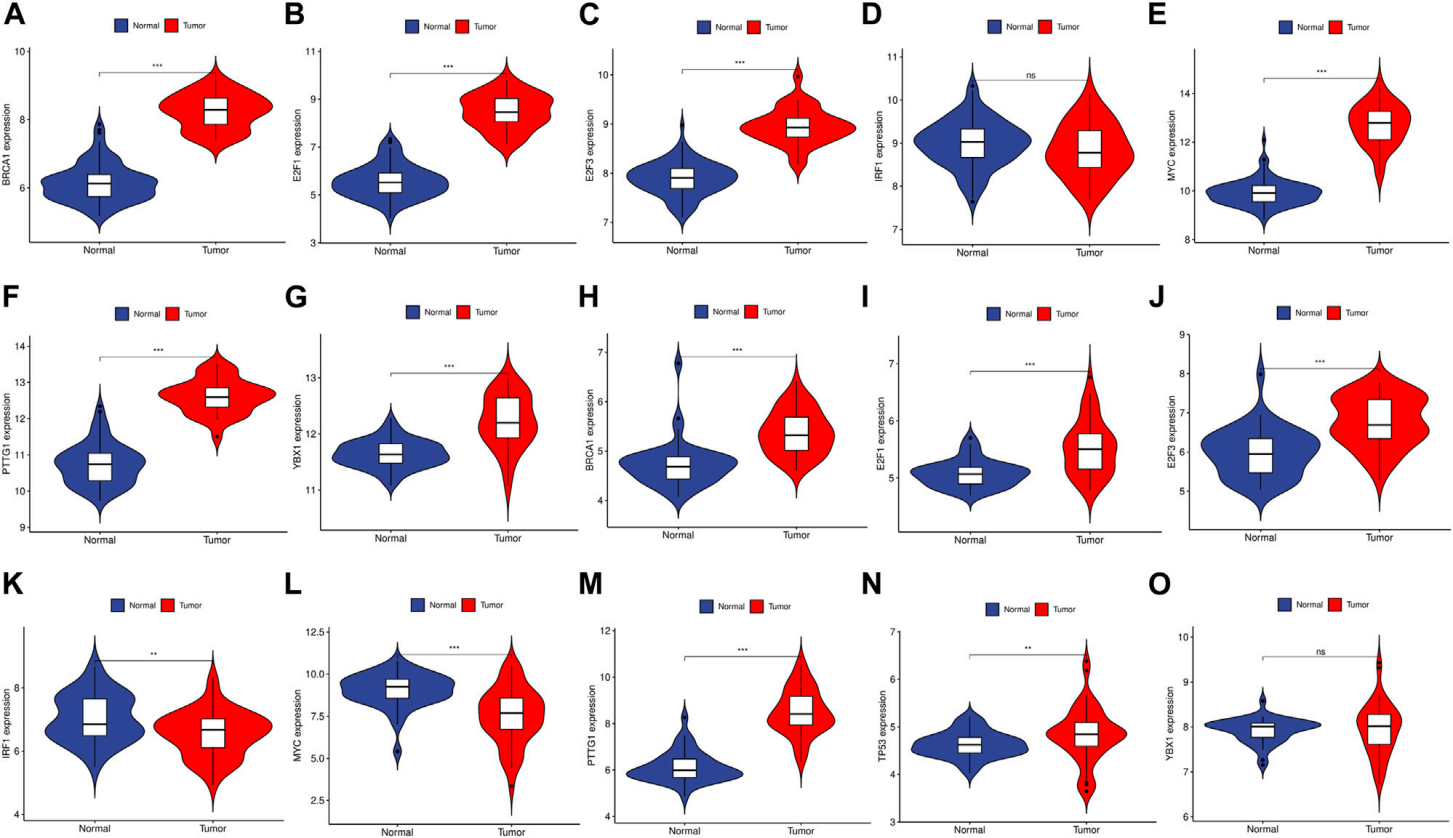
The expression level of TFs in GSE89076 and GSE50579. **(A–G)** The expression level of TFs in GSE89076. **(H–O)** The expression level of TFs in GSE50579. The upregulated genes are marked in light red; downregulated genes are marked in light blue. The comparison between the two sets of data uses the mean *t*-test. *p*-value <0.05 was considered statistically significant. *p < 0.05; **p < 0.01; ***p < 0.001.

We utilized the TRRUST database to identify 8 TFs that may regulate the expression of the aforementioned genes (Table 2; Figure 9). Additionally, we discovered that 6 of these TFs were highly expressed in both CRC and HCC, as demonstrated in Figures 10A–O. Furthermore, these TFs appeared to play a cooperative role in regulating the expression of 7 hub genes, including ASPM, CCNA2, CCNB1, CDC20, CDK1, MAD2L1, and TOP2A.

**TABLE 2:** Key transcriptional factors (TFs) of hub genes.

Key TFs	Description	*p*-value	Overlapped genes
E2F3	E2F transcription factor 3	3.06E-08	CCNB1,CDK1,CCNA2
YBX1	Y box binding protein 1	4.32E-07	TOP2A,CCNB1,CDC20
BRCA1	breast cancer 1, early onset	3.09E-06	ASPM, CCNB1,MAD2L1
PTTG1	pituitary tumor-transforming 1	2.64E-05	CCNB1,CDK1
E2F1	E2F transcription factor 1	4.05E-05	CCNB1,CDK1,TOP2A
TP53	tumor protein p53	7.39E-05	CCNA2,CCNB1,CDK1
IRF1	interferon regulatory factor 1	0.000318	CDK1,CCNB1
MYC	v-myc myelocytomatosis viral oncogene homolog (avian)	0.00122	CCNB1,CCNA2

## Discussion

Metastasis is thought to occur in multiple stages, starting with the primary tumor’s ability to survive in the bloodstream and enter the parenchyma of remote organs. Once there, the cancer cells must adapt to the foreign environment and grow into secondary tumors (Steeg, 2006). The ability to complete the metastatic process is thought to be limited to only a small number of tumor cells. Furthermore, the emergence of metastases occurs at varying rates, with certain organ sites being preferred depending on the type of cancer (Nguyen and Massagué, 2007; Chen et al., 2023; Liu et al., 2023). As a result, primary tumor cells and their metastatic descendants are expected to differ significantly in terms of tumor biology. While numerous genes associated with metastasis have been identified, their involvement in the development of metastasis in different types of tumors, their role in organ-specific metastasis, and how they drive progression are not yet fully understood (Sethi and Kang, 2011; Valastyan and Weinberg, 2011).

In recent years, the study and understanding of the tumor microenvironment has become as important as the modulation of cancer cell progression itself (Pavlova and Thompson, 2016; Sun et al., 2021; Ng et al., 2022). CRC and HCC are both highly resistant to therapy. Despite being the third leading cause of cancer-related death worldwide, HCC is not the only cancer type that is difficult to treat. Over the past decade, overall survival rates for CRC patients have not shown significant improvement (Torre et al., 2012; Fitzmaurice et al., 2015). Despite progress in available therapies, the survival rates of patients with CRC and HCC have only seen modest improvements. This is largely due to the lack of effective early detection methods and reliable prognostic indicators (Ye et al., 2015).

CRC and HCC might have overlapping pathogenic pathways. The mechanism of increased risk for multiple malignant tumors in patients with CRC is uncertain. Some etiologies, including underlying genetic background, lifestyle, and environmental risk factors, are involved with the development of cancers (Hemminki et al., 2001). In this study, we identified 148 overlapping DEGs in both, of which 10 were common hub genes, including CDK1, KIF11, CDC20, CCNA2, TOP2A, CCNB1, NUSAP1, BUB1B, ASPM and MAD2L1. According to our GO and KEGG Pathway enrichment analysis, the cell cycle regulation play an important role in these two diseases. Of these hub genes, CDK1, a kinase that acts on the core of the cell cycle, attracted our attention (Nurse and Bissett, 1981). The CDK1/cyclin B1 (CCNB1) complex is crucial not only for mitochondrial activities during cell cycle progression but also for the development of adaptive resistance in tumors (Wang et al., 2014; Liu et al., 2015). Proper regulation of cell proliferation is crucial for preventing the development of cancer as it plays a critical role in both the initiation and promotion of tumors (López-Sáez et al., 1998). The regulation and control of cancer cell proliferation are complex processes that involve numerous mechanisms, including those that accelerate or disrupt the cell cycle. Tumor suppressor pathways have been identified as negative regulators in signal transduction systems that lead to cell cycle arrest at various checkpoints (Mori et al., 1999). Significantly enriched differentially expressed genes that are positively associated with CDK1 were identified through publicly available expression profiles and KEGG and GO analyses. These genes were found to be involved in DNA replication, mismatch repair, and cell cycle pathways, indicating that CDK1 may interact with factors in these pathways to exert its effects.

Multiple studies have suggested that CDC20 plays a role in the oncogenesis of human cancers. Elevated CDC20 expression has been linked to clinicopathological features in various types of human cancers (Jeong et al., 2022). Elevated expression of CDC20 was observed in cell lines of colorectal cancer, with levels positively correlated with late clinical-stage, metastasis, and lower survival rates. These findings suggest that CDC20 could serve as a crucial diagnostic and prognostic biomarker for human colorectal cancer (Wu et al., 2013). In a previous study, CDC20 was found to be upregulated in 68% of HCCs compared to adjacent normal tissues. Notably, the overexpression of CDC20 was significantly associated with sex, tumor differentiation, and TNM stage (Li et al., 2014). These findings suggest that CDC20 may serve as a promising therapeutic target for HCC, although further investigations are needed to confirm the clinical implications of CDC20 suppression in the treatment of liver cancer.

Exploring the biological basis for differences in the prognostic value of hub genes in CRC and HCC is a key goal of this study. Of all the common hub genes, TOP2A is the most noteworthy because it has divergent survival outcomes in these two diseases. TOP2A is closely linked to cell proliferation and is therefore a promising therapeutic target for diseases characterized by increased cellular proliferation (Kim et al., 2015). TOP2A, a highly expressed gene in various types of carcinomas, is valuable in diagnosing cancer, monitoring disease progression, and predicting prognosis. In a previous study, TOP2A expression was assessed in 490 CRC tissues, and it was found that overexpression of TOP2A was associated with a lower T stage, lower N stage, and lower recurrence rate (Hua et al., 2015). Moreover, the study suggested that ZNF148 and TOP2A reciprocally regulate each other in CRC through the ceRNA mechanism involving miR101, miR144, miR335, and miR365, which is crucial in the regulation of cell proliferation in CRC (Gao et al., 2017). However, previous results show that TOP2A is involved in the development of liver cancer (Panvichian et al., 2015). In their report, Liu et al. demonstrated that MDM4 and TOP2A have a post-translational interaction, leading to their mutual upregulation and increased stability of TOP2A protein. This interaction also inhibits the p53 pathway and promotes tumor cell proliferation (Liu et al., 2019). In previous studies, the differential expression of MAD2L1 in many tumors was analyzed using the TIMER2.0 database (Chen et al., 2020). For CRC, the higher expression of MAD2L1 has been observed to counteract the repressive impact of miR-515-5p overexpression on the proliferation of colorectal cancer cells, as well as the induction of apoptosis and G1-phase detainment (Ding et al., 2022). However, survival analysis showed high expression of MAD2L1 in HCC correlated with shorter OS. This finding was also validated in other datasets which confirmed that high expression of MAD2L1 was an independent risk factor for OS in patients with HCC (Chen et al., 2022).

Current research is still inadequate to fully elucidate the distinct prognostic value of these hub genes in these two cancers, and further extensive investigations and clinical trials are warranted. The information on KEGs provides valuable insights into the pathogenesis of CRC and HCC, and could potentially aid in the development of targeted therapeutic approaches. Intriguingly, findings from a study on dual primary cancer involving both CRC and HCC indicated that the type, age of onset, interval of onset, and presence or absence of mutations in CRC-related genes in the first primary cancer did not significantly impact the prognosis of patients with dual primary cancer (Zhiping et al., 2023).

We analyzed related TFs and verified their expression levels in the original dataset, in addition to exploring hub genes. Our study provides potential directions for understanding the molecular mechanism of CRC complicated by HCC. Unlike previous studies (Cheng et al., 2020; Pan et al., 2022), we focused on common hub genes and related TFs due to the strong genetic similarity between the two cancers. To identify key nodes, we constructed a complex interaction network using their common DEGs. This comprehensive bioinformatics method has proven reliable in various diseases (Fang et al., 2020).

Advanced bioinformatics methods have not been extensively used to explore the shared molecular mechanisms between CRC and HCC. However, our research has some limitations, including its retrospective nature, which necessitates external validation of our findings. Additionally, the function of the identified hub gene requires further *in vitro* validation, which will be the focus of our future work. Nonetheless, our study has notable strengths. Given the high incidence of comorbidity between CRC and HCC, we identified common DEGs, hub genes, and TFs between these two cancers for the first time, thus providing further insights into their underlying mechanisms.

## Conclusion

Our study focused on identifying the shared DEGs in CRC and HCC. Furthermore, we conducted enrichment analysis and PPI network analysis. Our results revealed that CRC and HCC share numerous pathogenic pathways, which may be regulated by certain hub genes. This study presents novel perspectives that can aid in exploring the molecular mechanisms underlying the development of these two cancers.

## Data Availability

The datasets presented in this study can be found in online repositories. The names of the repository/repositories and accession number(s) can be found in the article/Supplementary Material.
