# Supplementary material for: Exploring the pathogenesis of colorectal carcinoma complicated with hepatocellular carcinoma via microarray data analysis
Source: Front Pharmacol. 2023 Jun 13;14:1201401. doi: 10.3389/fphar.2023.1201401 (PMC10293624; doi:10.3389/fphar.2023.1201401)
Supplement: Supplementary file 3 [file Table1.DOC]

| **Gene symbol** | **Type** |
| --- | --- |
| RACGAP1 | up |
| TTK | up |
| PRC1 | up |
| CCNB1 | up |
| CDKN3 | up |
| NDC80 | up |
| ECT2 | up |
| GINS1 | up |
| CENPW | up |
| STMN1 | up |
| BUB1B | up |
| RFC4 | up |
| TOP2A | up |
| CENPF | up |
| ASPM | up |
| STIL | up |
| AURKA | up |
| NEK2 | up |
| KIF4A | up |
| EZH2 | up |
| PTTG1 | up |
| MELK | up |
| RRM2 | up |
| PBK | up |
| DTL | up |
| HMMR | up |
| NUSAP1 | up |
| FLVCR1 | up |
| ZWINT | up |
| NUF2 | up |
| FOXM1 | up |
| HJURP | up |
| KPNA2 | up |
| UBAP2L | up |
| CDC20 | up |
| CDCA3 | up |
| KIAA0101 | up |
| DLGAP5 | up |
| UBE2C | up |
| CDK1 | up |
| SNRPD2 | up |
| BIRC5 | up |
| UHRF1 | up |
| ZWILCH | up |
| TACC3 | up |
| MKI67 | up |
| FANCI | up |
| UBE2T | up |
| BARD1 | up |
| TPX2 | up |
| TRIP13 | up |
| POLA1 | up |
| OIP5 | up |
| CENPM | up |
| CKS2 | up |
| MCM2 | up |
| MCM3 | up |
| CCDC34 | up |
| UBE2S | up |
| CDCA5 | up |
| MCM6 | up |
| CENPE | up |
| GPSM2 | up |
| MIS18A | up |
| HELLS | up |
| SPC25 | up |
| RNASEH2A | up |
| CCT6A | up |
| PRIM1 | up |
| ATAD2 | up |
| KIF11 | up |
| NPM1 | up |
| KIF2C | up |
| NUP155 | up |
| FAM83D | up |
| FEN1 | up |
| MAD2L1 | up |
| SMC4 | up |
| NCAPD2 | up |
| SMC2 | up |
| MSH2 | up |
| PODXL | up |
| FANCG | up |
| FLAD1 | up |
| TIPIN | up |
| MCM7 | up |
| VRK1 | up |
| CDC7 | up |
| BORA | up |
| PSPH | up |
| DBF4 | up |
| ITGB3BP | up |
| H2AFZ | up |
| SNRPB | up |
| PRR11 | up |
| RRP15 | up |
| CCNE2 | up |
| ACTL6A | up |
| CKS1B | up |
| KNTC1 | up |
| MSTO1 | up |
| TROAP | up |
| CDK4 | up |
| PEA15 | up |
| PRMT3 | up |
| DDX39A | up |
| LBR | up |
| OSBPL3 | up |
| TARBP1 | up |
| BLM | up |
| PAFAH1B3 | up |
| PCNA | up |
| DEPDC1 | up |
| OTUD6B | up |
| NME1 | up |
| SPIN4 | up |
| CEP55 | up |
| KIF23 | up |
| RPL39L | up |
| MICB | up |
| WDYHV1 | up |
| DNMT1 | up |
| LSM2 | up |
| DSN1 | up |
| C1orf112 | up |
| CCNA2 | up |
| CDC25B | up |
| SPAG5 | up |
| KIF15 | up |
| TK1 | up |
| PLP2 | up |
| CRNDE | up |
| SQLE | up |
| PRTFDC1 | up |
| MND1 | up |
| PSRC1 | up |
| IMPDH2 | up |
| LAPTM4B | up |
| IGF2BP3 | up |
| CDCA7 | up |
| LMNB1 | up |
| FLJ45482 | up |
| COL4A1 | up |
| S100P | up |
| HS6ST2 | up |
| FOXQ1 | up |
| MMP12 | up |
| LCN2 | up |
| HHIP | down |
| RSPO3 | down |
| NAT2 | down |
| CDHR2 | down |
| DNASE1L3 | down |
| VIPR1 | down |
| PLAC8 | down |
| FLJ22763 | down |
| DCN | down |
| RCAN1 | down |
| KBTBD11 | down |
| CXCL12 | down |
| MOGAT2 | down |
| GPR128 | down |
| GBA3 | down |
| SRPX | down |
| DPT | down |
| GSTZ1 | down |
| LILRB5 | down |
| IGF1 | down |
| ACAA2 | down |
| SOCS2 | down |
| MAN1C1 | down |
| ETFDH | down |
| FYN | down |
| MT1F | down |
| ACAA1 | down |
| EXPH5 | down |
| ZG16 | down |
| ASPG | down |
| MT1G | down |
| MT1M | down |
| CFHR3 | down |
| IL2RB | down |
| RNF125 | down |
| LDHD | down |
| CTBS | down |
| VSIG4 | down |
| CYP4F12 | down |
| CIDEB | down |
| GNE | down |
| MT1H | down |
| FAS | down |
| CYP3A4 | down |
| MT1X | down |
| PDE2A | down |
| CD163 | down |
| GABARAPL1 | down |
| PBLD | down |
| EPHX2 | down |
| ITLN1 | down |
| KLRB1 | down |
| MT1E | down |
| CLRN3 | down |
| NDRG2 | down |
| MT2A | down |
| FAM134B | down |
| MS4A7 | down |
| FOXO1 | down |
| DHRS1 | down |
| HMGCL | down |
| NAAA | down |
| SERPING1 | down |
| HBB | down |
| HCLS1 | down |
| EPB41L4B | down |
| ANG | down |
| CD14 | down |
| NTN4 | down |
| ACADS | down |
| PLSCR4 | down |
| GPT | down |
| IGJ | down |
| ALDH6A1 | down |
| SLC25A20 | down |
| CTSO | down |
| DUSP1 | down |
| CTH | down |
| ADH1A | down |
| UGP2 | down |
| SLC41A2 | down |
| PRKAR2B | down |
| XDH | down |
| SUCLG2 | down |
| MPEG1 | down |
| CCL19 | down |
| SULT1A2 | down |
| ADH1C | down |
| GRAMD1C | down |
| CSF1R | down |
| CPT2 | down |
| C1R | down |
| CCL21 | down |
| ABAT | down |
| LINC00261 | down |
| ACADSB | down |
| BDH1 | down |
| PPAP2B | down |
| TTR | down |
| NAT1 | down |
| CD69 | down |
| GIMAP4 | down |
| CD8A | down |
| CA2 | down |
| SLC46A3 | down |
| CDA | down |
| EVI2B | down |
| CCL5 | down |
| IL10RA | down |
| PPP1R3B | down |
| PON3 | down |
| NR3C2 | down |
| C1S | down |
| ADRB2 | down |
| MFAP4 | down |
| SCARNA17 | down |
| PCK1 | down |
| SLC1A1 | down |
| FMO4 | down |
| C1QA | down |
| GREM2 | down |
| TMEM56 | down |
| DAO | down |
| CYTIP | down |
| SMAD7 | down |
| HSD17B2 | down |
| ABCG2 | down |
| NOSTRIN | down |
| RAB26 | down |
| PXMP2 | down |
| RUNDC3B | down |
| CYP3A5 | down |
| HPGD | down |
| ARRDC4 | down |
| FABP1 | down |
| AGPAT9 | down |
| FBLN5 | down |
| PAPSS2 | down |
| PDK4 | down |
| CES2 | down |
| IL7R | down |
| RARRES3 | down |
| HGD | down |
| HMOX1 | down |
| SGK1 | down |
| HMGCS2 | down |
| KNG1 | down |
| PLA2G2A | down |
| CFH | down |
| IFIT1 | down |
